# The *Zymomonas mobilis *regulator *hfq *contributes to tolerance against multiple lignocellulosic pretreatment inhibitors

**DOI:** 10.1186/1471-2180-10-135

**Published:** 2010-05-07

**Authors:** Shihui Yang, Dale A Pelletier, Tse-Yuan S Lu, Steven D Brown

**Affiliations:** 1 Biosciences Division, Oak Ridge National Laboratory, Oak Ridge, TN, 37831, USA; 2 BioEnergy Science Center, Oak Ridge National Laboratory, Oak Ridge, TN, 37831, USA

## Abstract

**Background:**

*Zymomonas mobilis *produces near theoretical yields of ethanol and recombinant strains are candidate industrial microorganisms. To date, few studies have examined its responses to various stresses at the gene level. Hfq is a conserved bacterial member of the Sm-like family of RNA-binding proteins, coordinating a broad array of responses including multiple stress responses. In a previous study, we observed *Z. mobilis *ZM4 gene ZMO0347 showed higher expression under anaerobic, stationary phase compared to that of aerobic, stationary conditions.

**Results:**

We generated a *Z. mobilis hfq *insertion mutant AcRIM0347 in an acetate tolerant strain (AcR) background and investigated its role in model lignocellulosic pretreatment inhibitors including acetate, vanillin, furfural and hydroxymethylfurfural (HMF). *Saccharomyces cerevisiae *Lsm protein (Hfq homologue) mutants and Lsm protein overexpression strains were also assayed for their inhibitor phenotypes. Our results indicated that all the pretreatment inhibitors tested in this study had a detrimental effect on both *Z. mobilis *and *S. cerevisiae*, and vanillin had the most inhibitory effect followed by furfural and then HMF for both *Z. mobilis *and *S. cerevisiae*. AcRIM0347 was more sensitive than the parental strain to the inhibitors and had an increased lag phase duration and/or slower growth depending upon the conditions. The *hfq *mutation in AcRIM0347 was complemented partially by trans-acting *hfq *gene expression. We also assayed growth phenotypes for *S. cerevisiae *Lsm protein mutant and overexpression phenotypes. Lsm1, 6, and 7 mutants showed reduced tolerance to acetate and other pretreatment inhibitors. *S. cerevisiae *Lsm protein overexpression strains showed increased acetate and HMF resistance as compared to the wild-type, while the overexpression strains showed greater inhibition under vanillin stress conditions.

**Conclusions:**

We have shown the utility of the pKNOCK suicide plasmid for mutant construction in *Z. mobilis*, and constructed a Gateway compatible expression plasmid for use in *Z. mobilis *for the first time. We have also used genetics to show *Z. mobilis *Hfq and *S. cerevisiae *Lsm proteins play important roles in resisting multiple, important industrially relevant inhibitors. The conserved nature of this global regulator offers the potential to apply insights from these fundamental studies for further industrial strain development.

## Background

Biomass-based bioenergy is crucial to meet national goals of making cellulosic ethanol cost-competitive with gasoline. A core challenge in fermenting cellulosic material to ethanol is the recalcitrance of biomass to breakdown. Severe biomass pretreatments are therefore required to release the sugars, which along with by-products of fermentation can create inhibitors including sugar degradation products such as furfural and hydroxymethylfurfural (HMF); weak acids such as acetic, formic, and levulinic acids; lignin degradation products such as the substituted phenolics vanillin and lignin monomers [1]. In addition, the metabolic byproducts such as ethanol, lactate, and acetate also influence the fermentation by slowing and potentially stopping the fermentation prematurely. The increased lag phase and slower growth increases the ethanol cost due to both ethanol production rate and total ethanol yield decreases [2,3]. One approach to overcome the issue of inhibition caused by pretreatment processes is to remove the inhibitor after pretreatment from the biomass physically or chemically, which requires extra equipment and time leading to increased costs. A second approach utilizes inhibitor tolerant microorganisms for efficient fermentation of lignocellulosic material to ethanol and their utility is considered an industrial requirement [1].

*Z. mobilis *are Gram-negative facultative anaerobic bacteria with a number of desirable industrial characteristics, such as high-specific productivity and ethanol yield, unique anaerobic use of the Entner-Doudoroff pathway that results in low cell mass formation, high ethanol tolerance (12%), pH 3.5-7.5 range for ethanol production and has a generally regarded as safe (GRAS) status [4-9]. *Z. mobilis *strains have been engineered to ferment pentose sugars such as xylose, arabinose and other substrates with high yields, but a low tolerance to acetic acid and decreased ethanol tolerance have been reported in recombinant strains [4,10-12]. *Z. mobilis *mutant strains tolerant to a pretreatment inhibitor such as acetate have been generated by chemical mutagenesis with *N*-methyl *N*'-nitro *N*-nitrosoguanidine and selection in continuous culture with a progressively increasing concentration of sodium acetate in the medium feed [13]. AcR is capable of efficient ethanol production in the presence of 20 g/L NaAc, while the parent ZM4 is inhibited significantly above 12 g/L NaAc under the same conditions [13].

We have investigated *Z. mobilis *ZM4 gene expression and metabolomic profiles during aerobic and anaerobic conditions and found that aerobic, stationary phase conditions produced a number of inhibitory secondary metabolites [14] and the expression of a putative *hfq *gene ZMO0347 was greater in anaerobic stationary phase compared to that of aerobic conditions [14]. Hfq is a global regulator that acts as an RNA chaperone and is involved in coordinating regulatory responses to multiple stresses [15-18]. However, little is known about *Z. mobilis *Hfq.

The aim of this study was to investigate the role of a putative *hfq *gene ZMO0347 on multiple pretreatment inhibitor tolerances. *Z. mobilis *genetic modification has been reported previously with the *sacC*, *adhB*, and *ndh *targets for mutagenesis [19-21]. However, the existence of native plasmids [22,23] and intrinsic antibiotic resistance impedes the use of many broad-host-range plasmids [22,24,25]. In this work, we identified appropriate antibiotics for *Z. mobilis *genetic studies, created an expression plasmid vector, and utilized the pKNOCK-Km suicide plasmid [26] to create an *hfq *mutant in *Z. mobilis *acetate tolerant strain AcR. We demonstrate that the *Z. mobilis hfq *is important for *Z. mobilis *tolerance to several classes of lignocellulosic pretreatment inhibitors.

Hfq is part of an ancient family of proteins termed Sm and Sm-like (Lsm) proteins that are conserved among bacteria, archaea, and eukaryotes such as yeast *S. cerevisiae *[16,27]. Seven core yeast Sm proteins form a heteroheptameric ring with a small central hole and are essential [28]. Eight Lsm proteins (LSM1, LSM2, LSM3, LSM4, LSM5, LSM6, LSM7, and LSM8) in *S. cerevisiae *form two different heteroheptameric rings containing either Lsm1p or Lsm8p with common Lsm2p-7p components [28]. The complex containing Lsm2-8p localizes to the nucleus and is involved in nuclear RNA processing, and the complex containing Lsm1-7p contributes to cytoplasmic RNA processing [28,29]. In addition, LSM9 (MAK31) has also been reported to contain a Sm domain, as well as other proteins such as LSM12 (YHR121W), LSM13 (SCD6, YPR129W), and LSM16 (EDC3, YEL015W) [29]. In this study, we also show that *S. cerevisiae *Lsm1, 6, and 7 proteins contribute to yeast pretreatment inhibitor tolerance.

## Results

### Bioinformatics analysis of a putative *Z. mobilis *Hfq and related *S. cerevisiae *proteins

To assess whether *Z. mobilis *ZMO0347 was similar to other known members of the Hfq family of regulators, the ZMO0347 protein sequence was used in a BlastP analysis [30]. The BlastP result indicates that ZMO0347 is similar to the *E. coli *global regulator Hfq protein (60% sequence identity), and to eukaryotic homologues such as Sm or Lsm proteins exist in other microorganisms like *S. cerevisiae *(Additional file 1). These analyses suggest that ZMO0347 is likely an Hfq regulator family protein in *Z. mobilis*. Interestingly, the *Z. mobilis *ZMO0347 (Hfq) protein possesses two Sm-like family domains, two intra-hexamer interaction sites, two inter-hexamer interaction sites, two nucleotide binding pockets, and has an extra Sm-like domain near the C-terminus (Additional file 1A) which is unlike most of the bacterial Hfq protein sequences that have only one Sm-like domain (Additional file 1). *S. cerevisiae *has nineteen proteins with a Sm or Sm-like domain, and although examples like Sm protein (SmB) and Lsm protein (Lsm1) (Additional file 1C, D, respectively) contain Sm-like domains, significant sequence similarity was not observed by BlastP analysis.

### *Z. mobilis *AcR strain *hfq *mutant construction and complementation

Intrinsic *Z. mobilis *antibiotic resistance has been reported previously [22,25], which restricts the use of the available broad-host-range plasmids. We tested the antibiotic sensitivities of ZM4 and AcR as an initial step for genetic studies with these strains. Each strain was tested against the following antibiotics; chloramphenicol (25, 50, 100, and 200 μg/mL), gentamicin (100, 200, and 300 μg/mL), kanamycin (100, 200, and 300 μg/mL), streptomycin (200 and 300 μg/mL), and tetracycline (25, 50, 100, and 200 μg/mL). Each assay was conducted under aerobic and anaerobic conditions and similar growth results were observed under the respective conditions for the different doses. *Z. mobilis *was tolerant to streptomycin at concentration of 300 μg/mL and gentamicin at 100 μg/mL. *Z. mobilis *was able to grow slightly at 100 μg/mL kanamycin and 300 μg/mL gentamicin, and was sensitive to tetracycline and chloramphenicol at concentrations above 25 μg/mL (data not shown).

We generated an *hfq *insertion mutant in a *Z. mobilis *acetate tolerant strain (AcR) background using the pKnock-Km suicide plasmid system [26,31], and designated it as strain AcRIM0347 (See Methods for details). Since many mutagenesis systems use either chloramphenicol or kanamycin markers, tetracycline resistance was used as an expression plasmid antibiotic marker for new Gateway entry vector pBBR3DEST42 construction (Additional file 2), which was then used to generate plasmid p42-0347 to express *hfq *gene ZMO0347. The nucleotide sequence for plasmid p42-0347 was verified by Sanger sequencing, and the expression of *hfq *from plasmid p42-0347 in *E. coli *was confirmed by Western blot analysis (data not shown). Plasmid p42-0347 was then introduced into the wild-type strain ZM4, acetate tolerant mutant AcR, and *hfq *mutant AcRIM0347 background by conjugation and selection. The presence of correct p42-0347 construct in ex-conjugants was confirmed by PCR and Sanger sequencing analysis. The oligonucleotide primers used to generate *hfq *mutant AcRIM0347 and complement *hfq *mutation in AcRIM0347 are listed in Table 1.

**Table 1:** Bacterial strains, plasmids and primers used in this study

Strain, plasmid, or primer	Genotype, phenotype, or sequence of primer (5' to 3')	Reference
***E. coli***		
K-12	K-12 MG1655 Wild-type strain	
DH5α	F^- ^ψ80d*lac*ZΔM15 Δ(*lac*ZYA-*arg*F) U169 *rec*A1 *end*A1 *hsd*R17(r_k_^-^, m_k_^+^) *pho*A *sup*E44 λ^- ^*thi*-1 *gyr*A96 *rel*A1	Novagen
DB3.1	F^- ^*gyr*A462 *end*A1Δ(sr1-*rec*A) *mcr*B *mrr hsd*S20(r_B_^-^, m_B_^-^) *sup*E44 *ara*-14 *gal*K2 *lac*Y1 *pro*A2 *rps*L20(Sm^R^) *xyl*-5λ-l*eu mtl*1	Invitrogen
WM3064	*thrB1*004 *pro thi rpsL hsdS lacZ*Δ*M15 *RP4-1360 Δ(*araBAD)567 *Δ*dapA*1341::[*erm pir*]	W. Metcalf
BL21(DE3)	F- *omp*T *hsd*SB(rB-mB-) *gal dcm *(DE3)	Invitrogen

***Z. mobilis***		
ZM4	ATCC31821	ATCC
AcR	ZM4 acetate tolerant strain generated by random mutagenesis	[13]
AcRIM0347	*hfq*::pKm-0347, insertional mutant of AcR *hfq *gene ZMO0347	This study
AcRIM0347 (p42-0347)	AcRIM0347 containing plasmid p42-0347	This study
ZM4(p42-0347)	ZM4 containing plasmid p42-0347	This study
AcR (p42-0347)	AcR containing plasmid p42-0347	This study

***S. cerevisiae***		
BY4741	MATa his3Δ1 leu2Δ0 ura3Δ0 met15Δ0 - s288c background	Open Biosystems
YSC1021-554440	Yeast: Yeast Knock Out Strain (YKO_LSM1) Clone Id: 1301; Accession: YJL124C (Lsm1)	Open Biosystems
YSC1021-552226	Yeast: Yeast Knock Out Strain (YKO_LSM6) Clone Id: 4214; Accession: YDR378C (Lsm6)	Open Biosystems
YSC1021-556031	Yeast: Yeast Knock Out Strain (YKO_LSM7) Clone Id: 7383; Accession: YNL147W (Lsm7)	Open Biosystems
YSC1021-552677	Yeast: Yeast Knock Out Strain (YKO_LSM9) Clone Id: 3501; Accession: YCR020C-A (Mak31P, Lsm9)	Open Biosystems
YSC1021-552563	Yeast: Yeast Knock Out Strain (YKO_LSM12) Clone Id: 1949; Accession: YHR121W (Lsm12)	Open Biosystems
YSC1021-553518	Yeast: Yeast Knock Out Strain (YKO_LSM13) Clone Id: 5544; Accession: YPR129W (Lsm13)	Open Biosystems
YSC1021-552280	Yeast: Yeast Knock Out Strain (YKO_LSM16) Clone Id: 255; Accession: YEL015W (Lsm16)	Open Biosystems
YSC4515-98807049	Yeast GST-Tagged Strain (GST_LSM1) Clone Id: YJL124C; Accession: YJL124C (lsm1)	Open Biosystems
YSC4515-98811389	Yeast GST-Tagged Strain (GST_LSM6) Clone Id: YDR378C; Accession: YDR378C (Lsm6)	Open Biosystems
YSC4515-98805426	Yeast GST-Tagged Strain (GST_LSM9) Clone Id: YCR020C-A; Accession: YCR020C-A (Mak31P, Lsm9)	Open Biosystems
YSC4515-98806813	Yeast GST-Tagged Strain (GST_LSM12) Clone Id: YHR121W; Accession: YHR121W (Lsm12)	Open Biosystems
YSC4515-98808930	Yeast GST-Tagged Strain (GST_LSM13) Clone Id: YPR129W; Accession: YPR129W (Lsm13)	Open Biosystems
YSC4515-98809076	Yeast GST-Tagged Strain (GST_LSM16) Clone Id: YEL015W; Accession: YEL015W (Lsm16)	Open Biosystems

**Plasmids**		
pKNOCK-Km	Km^r^, *mob*, broad host range suicide cloning vector, 1.8 kb	[26]
pET-DEST42	Ap^r^, Cm^r^, C-terminal 6×His and V5 epitope	Invitrogen
pDONR221	Km^r^, gateway entry vector Gm^r^, N-terminal GST	Invitrogen
pBBR1MCS-3	Tc^r^, *mob*, broad host range cloning vector	[36]
pBBR3DEST42	Cm^r ^Tc^r^, C-terminal 6×His and V5 epitope	This study
pKm-0347	pKnock-Km containing 262-bp *hfq *internal fragment for insertional mutant construction	This study
p42-0347	pBBR3DEST42 containing ZM4 gene ZMO0347	This study

**PCR Primers**		
hfq_MF	cggagagatggtcagtcaca	262-bp
hfq_MR	ttcttgctgctgcataatcg	

hfq_CF	ggggacaagtttgtacaaaaaagcaggcttcgaaggagatagaATGGCCGAAAAGGTCAACAATC	483-bp
hfq_CR	ggggaccactttgtacaagaaagctgggtcATCCTCGTCTCGGCTTTCTG	

hfq_OCF	Caaagcttgagctcgaattcatttttgccgtggtagttgc	1050-bp
hfq_OCR	caggtacctctagaattcaccactcaatcctcgtctcg	

hfq_MF and hfq_MR are primers used for insertional mutant construction using pKnock mutagenesis system. Hfq_OCF and Hfq_OCR are primers for mutant confirmation. Hfq_CF and Hfq_CR are primers used to clone the *hfq *gene into low copy number Gate-Way compatible plasmid pBBR3DEST42 for complementation, which results in a plasmid called p42-0347.

### *Z. mobilis hfq *contributes to pretreatment inhibitor tolerance

**Figure 1:**
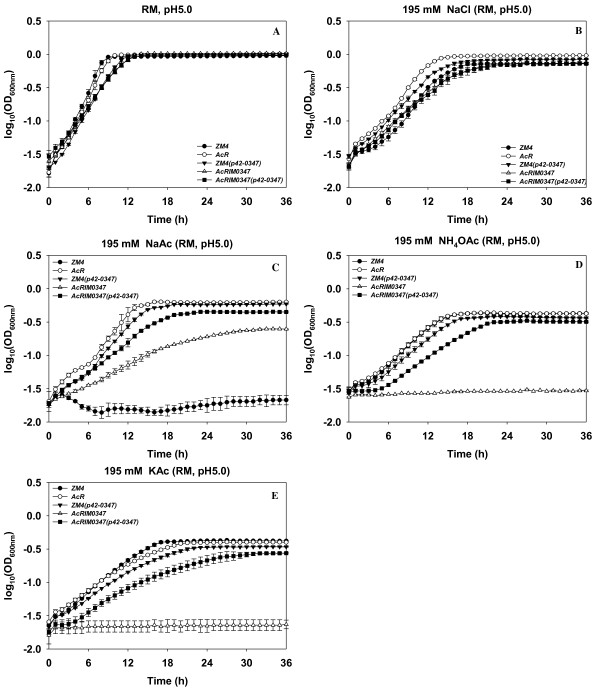
**Hfq contributes to *Z. mobilis *acetate tolerance**. *Z. mobilis *strains were grown in RM (pH5.0) overnight, 5-μL culture were then transferred into 250-μL RM media in the Bioscreen plate. The growth differences of different strains were monitored by Bioscreen (Growth Curves USA, NJ) under anaerobic conditions; in RM, pH 5.0 (A), RM with 195 mM NaCl, pH 5.0 (B), 195 mM NaAc, pH 5.0 (C), 195 mM NH_4_OAc, pH 5.0 (D), or 195 mM KAc, pH 5.0 (E). Strains included in this study are: ZM4: *Zymomonas mobilis *ZM4 wild-type; AcR: previously described ZM4 acetate tolerant mutant; ZM4 (p42-0347): ZM4 containing a gateway plasmid p42-0347 to express ZM4 gene ZMO0347; AcRIM0347: AcR insertional mutant of ZMO0347; AcRIM0347 (p42-0347): AcRIM0347 containing gateway plasmid p42-0347. This experiment has been repeated at least three times with similar result. Duplicate biological replicates were used for each condition.

**Figure 2:**
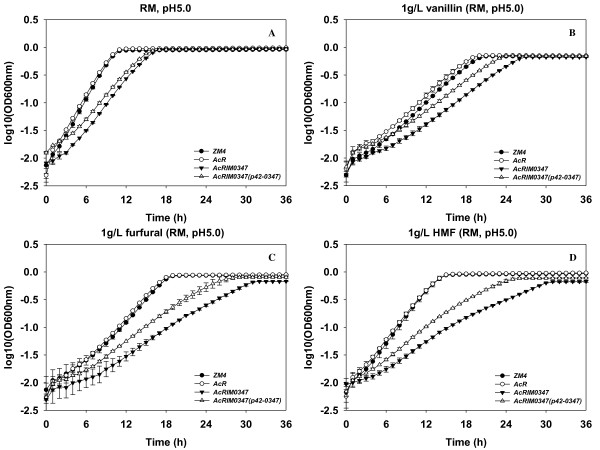
***Z. mobilis *tolerance to different classes of pretreatment inhibitors and Hfq**. *Z. mobilis *strains were grown in RM (pH 5.0) overnight, 5-μL culture were then transferred into 250-μL RM media in the Bioscreen plate. The growth differences of different strains were monitored by Bioscreen (Growth Curves USA, NJ) under anaerobic conditions in RM, pH 5.0 (A), RM with 1 g/L vanillin, pH 5.0 (B), 1 g/L furfural, pH 5.0 (C), and 1 g/L HMF, pH 5.0 (D).

**Pretreatment inhibitors had negative effects on *Z. mobilis *growth**: the growth of *Z. mobilis *strains was reduced in the presence of acetate, vanillin, furfural, or HMF with increased lag phases and/or slower growth rates and/or final bacterial cell densities depending on the respective condition and strain (Table 2, 3; Fig. 1, 2). Among the different forms of acetate counter-ions tested, sodium acetate had the most inhibitory effect on wild-type *Z. mobilis *growth. This was followed by potassium acetate and ammonium acetate and sodium chloride had the least negative influence on wild-type *Z. mobilis *growth (Table 2; Fig. 1). Wild-type ZM4 growth was completely inhibited when RM medium was amended with 195 mM sodium acetate (Table 2; Fig. 1C) in keeping with previous reports [13]. Among the pretreatment inhibitors of vanillin, furfural, and HMF, vanillin had the most inhibitory effect on *Z. mobilis *and HMF the least (Table 3). *Z. mobilis *took longer to complete active growth and reach the stationary phase, which was about 16, 19 or 21 h in the presence of HMF, furfural or vanillin, respectively, compared to 11 h without any inhibitor present in the medium (Fig. 2).

**Table 2:** Growth rate and final cell density of different *Z. mobilis *strains in the absence or presence of different sodium and acetate ions.

		ZM4	AcR	AcRIM0347	AcRIM0347 (p42-0347)	ZM4 (p42-0347)
**Growth rate (hour^-1^)**	**RM**	0.42 ± 0.01	0.39 ± 0.01	0.32 ± 0.003	0.33 ± 0.002	0.38 ± 0.003
	**RM (NaCl)**	0.24 ± 0.008	0.29 ± 0.005	0.21 ± 0.008	0.22 ± 0.009	0.25 ± 0.008
	**RM (NH_4_OAc)**	0.20 ± 0.008	0.19 ± 0.005	NA	0.22 ± 0.002	0.19 ± 0.007
	**RM (Kac)**	0.15 ± 0.004	0.12 ± 0.000	NA	0.09 ± 0.003	0.12 ± 0.006
	**RM (NaAc)**	NA	0.29 ± 0.04	0.12 ± 0.004	0.16 ± 0.002	0.27 ± 0.004

**Final Cell Density (OD_600 nm_)**	**RM**	0.95 ± 0.006	1.01 ± 0.006	0.94 ± 0.004	0.92 ± 0.002	1.02 ± 0.004
	**RM (NaCl)**	0.73 ± 0.01	0.96 ± 0.01	0.73 ± 0.03	0.72 ± 0.02	0.84 ± 0.01
	**RM (NH_4_OAc)**	0.43 ± 0.01	0.42 ± 0.006	NA	0.32 ± 0.007	0.37 ± 0.008
	**RM (Kac)**	0.42 ± 0.002	0.40 ± 0.000	NA	0.28 ± 0.007	0.34 ± 0.004
	**RM (NaAc)**	NA	0.63 ± 0.02	0.25 ± 0.001	0.45 ± 0.002	0.59 ± 0.002

**"NA**" indicates that the data are not available due to the lack of growth in that condition. The concentration for all the chemicals (NaCl, NH_4_OAc, KAc, NaAc) supplemented into the RM is 195 mM. NaCl: sodium chloride, NH_4_OAc: ammonium acetate, KAc: potassium acetate, NaAc: sodium acetate. Strains included in this study are: ZM4: *Zymomonas mobilis *ZM4 wild-type; AcR: previously described ZM4 acetate tolerant mutant; ZM4 (p42-0347): ZM4 containing a gateway plasmid p42-0347 to express ZM4 gene ZMO0347; AcRIM0347: AcR insertional mutant of ZMO0347; AcRIM0347 (p42-0347): AcRIM0347 containing gateway plasmid p42-0347. This experiment has been repeated at least three times with similar result. Duplicate biological replicates were used for each condition.

**Table 3:** Growth rate and final cell density of different *Z. mobilis *strains in the absence or presence of different pretreatment inhibitors.

		ZM4	AcR	AcRIM0347	AcRIM0347(p42-0347)
**Growth rate (hour^-1^)**	**RM**	0.48 ± 0.03	0.46 ± 0.003	0.35 ± 0.004	0.32 ± 0.003
	**HMF**	0.36 ± 0.02	0.35 ± 0.01	0.19 ± 0.02	0.22 ± 0.001
	**Furfural**	0.31 ± 0.01	0.30 ± 0.005	0.19 ± 0.03	0.20 ± 0.01
	**Vanillin**	0.26 ± 0.001	0.26 ± 0.01	0.20 ± 0.006	0.20 ± 0.003

**Final Cell Density (OD_600 nm_)**	**RM**	0.91 ± 0.01	0.98 ± 0.006	0.95 ± 0.003	0.92 ± 0.006
	**HMF**	0.93 ± 0.003	0.96 ± 0.006	0.67 ± 0.03	0.78 ± 0.02
	**Furfural**	0.88 ± 0.006	0.89 ± 0.009	0.67 ± 0.001	0.80 ± 0.02
	**Vanillin**	0.69 ± 0.006	0.71 ± 0.01	0.66 ± 0.01	0.70 ± 0.01

The concentration for the inhibitor supplemented into the RM is: HMF: 0.75 g/L, furfural, or vanillin: 1 g/L. Strains included in this study are: ZM4: *Zymomonas mobilis *ZM4 wild-type; AcR: previously described ZM4 acetate tolerant mutant; AcRIM0347: AcR insertional mutant of ZMO0347; AcRIM0347 (p42-0347): AcRIM0347 containing gateway plasmid p42-0347. This experiment has been repeated at least three times with similar result. Duplicate biological replicates were used for each condition.

**Hfq contributes to sodium and acetate ion tolerances**: although the final cell density of *hfq *mutant AcRIM0347 is similar to that of AcR parental strain (Table 2; Fig. 2A), the growth rate of AcRIM0347 was reduced about one-fifth even without any inhibitor in the RM, which indicates *hfq *plays a central role in normal *Z. mobilis *physiology. Wild-type ZM4 that contained p42-0347 was able to grow in the presence of 195 mM sodium acetate and had a similar growth rate and final cell density to that of acetate tolerant strain AcR (Table 2; Fig. 1C). The wild-type ZM4 was unable to grow under this condition.

The inactivation of the *hfq *gene in AcR decreased this acetate tolerant strain's resistance to both sodium ion (sodium chloride) and acetate ion (ammonium acetate and potassium acetate) (Table 2; Fig. 1). *hfq *mutant AcRIM0347 was unable to grow in the presence of 195 mM ammonium acetate or potassium acetate (Table 2; Fig. 1D, E). Both the growth rate and final cell density of *hfq *mutant AcRIM0347 were reduced by at least a quarter in the presence of 195 mM sodium chloride, and about 60% in the presence of 195 mM sodium acetate compared to that of the parental strain AcR (Table 2; Fig. 1B, C). The AcRIM0347 *hfq *mutation was complemented by the introduction of an *hfq*-expressing plasmid (p42-0347) into the strain. The complemented mutant strain recovered at least half of the parental strains growth rate and 70% of its final cell density in the presence of 195 mM acetate ion (whether as sodium, ammonium or potassium acetate) (Table 2; Fig. 1).

**Hfq contributes to vanillin, furfural and HMF tolerances**: AcRIM0347 growth rates were lower than that of ZM4 and AcR under all conditions tests, and except for growth in RM broth (Table 3; Fig. 2). AcRIM0347 also achieved lower final cell densities compared to ZM4 and AcR (Table 3; Fig. 2). When AcRIM0347 was provided functional *Z. mobilis *Hfq via p42-0347, growth rates under all conditions were largely unchanged (Table 3). However, shorter lag phases were observed for AcRIM0347 (p42-0347) grown with vanillin, furfural or HMF and increases in final cell densities were also observed under these conditions (Table 3; Fig. 2). These data indicate that *hfq *is important for optimal *Z. mobilis *growth and its ability to resist furfural, HMF and vanillin toxicity.

### Yeast Lsm proteins contribute to pretreatment inhibitor tolerance

#### Lsm protein and yeast tolerance to sodium and acetate ions

*S. cerevisiae *Sm and Sm-like (Lsm) proteins are similar to *Z. mobilis *Hfq at the level of protein sequence (Additional file 1). Growth of yeast Lsm deletion mutants and Lsm over-expressing strains in 305 mM ammonium acetate, potassium acetate, or sodium acetate was assessed to test whether *S. cerevisiae *Lsm proteins and ZM4 Hfq had functionally similar roles. These studies included seven Lsm deletion mutants affecting three Lsm heteroheptameric ring components (Lsm1, Lsm6, Lsm7) and four other Lsm proteins containing an Sm domain (Lsm9, Lsm12, Lsm13, Lsm16), as well as six Lsm protein over-expressing strains (Lsm1, Lsm6, Lsm9, Lsm12, Lsm13, Lsm16). We present growth data for those genes that gave clear phenotypic differences for the sake of clarity and a list of all strains tested in this study can be found in Table 1.

Growth differences between the Lsm mutants and yeast wild-type BY4741 in the CM broth without the addition of acetate or with 305 mM NaCl were not observed (Additional file 3A, B, respectively). *S. cerevisiae *Lsm proteins involved in RNA processing ring complex formation (Lsm1, 6, 7), especially Lsm6, played a role in acetate tolerance (Additional file 3C-E, K-M). Lsm protein deletion mutants Lsm1, 6, and 7 showed decreased acetate tolerance compared to the wild-type control strain, especially in early growth stages for acetate with sodium, ammonium and potassium counter-ions (Additional file 3C-E). The Lsm overexpression strains grew similarly to wild-type BY4741 without the addition of acetate or with 305 mM NaCl (Additional file 3I, J), but each of the Lsm protein overexpression strains showed enhanced acetate tolerance compared to the wild-type strain with sodium, ammonium or potassium counter-ions (Additional file 3K-M).

#### Lsm proteins and yeast tolerance to vanillin, furfural and HMF

the effect of Lsm proteins on *S. cerevisiae *tolerance to pretreatment inhibitors vanillin, furfural, and HMF was also investigated using the seven Lsm deletion mutants and six Lsm overexpression strains described above. Each yeast deletion mutant and each overexpression strain showed similar growth profiles compared to wild-type strain BY4741 in the absence of inhibitors (Additional file 3A; I). Deletion mutants for Lsm1, 6 and 7 proteins were unable to grow or showed extended lag phases before recovery from the inhibitory effects of pretreatment inhibitors (Additional file 3F-H). Overexpression of Lsm proteins provided a slight growth advantage in the presence of 1.5 g/L HMF and furfural (Additional file 3O-P). However, a detrimental effect on growth was observed for overexpression strains when cultured in the presence of 0.75 g/L vanillin (Additional file 3N). The data indicated that Lsm proteins Lsm1, 6, and 7 especially Lsm6, which are the components of yeast RNA processing ring complex, play a role in tolerance to the model inhibitors used in this study.

## Discussion

A previous study indicated that *Z. mobilis *ZM4 *hfq *was less abundant in aerobic, stationary phase fermentations compared to the equivalent anaerobic condition and that *rpoH *was induced under the aerobic condition [14]. The role of *Z. mobilis *regulators like Hfq and extent of cross talk between regulatory networks remains to be elucidated. This study indicated that *hfq *also plays a role in *Z. mobilis *resistance to both acetate (sodium acetate, potassium acetate, or ammonium acetate) and sodium ions (sodium chloride and sodium acetate) (Table 2; Fig. 1). A recent study has identified that *nhaA *overexpression (encoding a sodium-proton antiporter) conferred the previously reported AcR (sodium acetate tolerant) mutant phenotype [32]. Constitutive *nhaA *over-expression in strain AcRIM0347 (*hfq*^-^) is a likely possibility for it being unable to survive with 195 mM ammonium acetate or potassium acetate, while the same concentration of sodium acetate only partially repressed its growth. *hfq *or *nhaA *each contribute to sodium acetate tolerance (Table 2; Fig. 1C) [32], but there is no additive benefit for increased inhibitor tolerance for *hfq *and *nhaA *if both were over-expressed at the same time (data not shown). In addition, the overexpression of *nhaA *gene in *Z. mobilis *had no advantage over other physiological stress responses for model pretreatment inhibitors such as vanillin, furfural, and HMF [32]. While *Z. mobilis hfq *contributes to the tolerance of these inhibitors as shown by increased *hfq *mutant AcRIM0347 lag phases and slower growth rates during early logarithmic growth phase compared to AcR strain (Fig. 2). These separate studies indicate there may often be more than one pathway for industrial strain development.

The majority of proteins similar to *Z. mobilis *Hfq contained one Sm-like superfamily domain (Additional file 3), with the exception of those from six other species also within the Sphingomonadales. Future structural studies are required to define the role for *Z. mobilis *and other microorganisms with two Sm-like family domains, to elucidate Hfq subunit interactions, and to test whether only three Hfq proteins would be needed for *Z. mobilis *to form the active homo-hexameric ring structure.

We assayed growth phenotypes for *S. cerevisiae *Lsm protein mutant and overexpression phenotypes. Lsm1, 6, and 7 mutants showed reduced tolerance to acetate and other pretreatment inhibitors (Additional file 3). The *S. cerevisiae *Lsm over-expression studies showed these strains had increased acetate and HMF resistance compared to the wild-type strain, while the overexpression strains were more inhibited under vanillin stress conditions (Additional file 3). The conserved nature of Sm-like proteins, the involvement of ZM4 Hfq and *S. cerevisiae *Sm-like proteins in pretreatment inhibitor tolerance offers the possibility of manipulating *hfq *and members of the Lsm family for strain development purposes.

## Conclusions

We created a plasmid for gene expression and mutation complementation in *Z. mobilis *and used the pKnock system to create an *hfq *mutant in *Z. mobilis *acetate tolerant strain AcR. We showed that *Z. mobilis hfq *played a role in tolerance to multiple biomass pretreatment inhibitors including acetate, vanillin, furfural, and HMF. In addition, Hfq homologues of yeast Lsm proteins Lsm1, 6, and 7 involving in the RNA processing heteroheptameric ring complex formation, especially Lsm6, contribute to multiple pretreatment inhibitor tolerance in *S. cerevisiae*. However, further studies such as systems biology studies and ChIP-Seq are required to elucidate the *hfq *stress response regulon in *Z. mobilis *and the yeast inhibitor tolerance genes affected by the RNA processing Lsm complexes.

## Methods

### Strains and culture conditions

Bacterial strains and plasmids used in this study are listed in Table 1. *E. coli *strains were cultured using Luria-Bertani (LB) broth or agar plates. *E. coli *WM3064 was supplemented with 100 μg/mL diaminopimelic acid (DAP). *Z. mobilis *ZM4 was obtained from the American Type Culture Collection (ATCC 31821) and the *Z. mobilis *acetate tolerant strain AcR has been described previously [13]. ZM4 and AcR were cultured in RM medium (Glucose, 20.0 g/L; Yeast Extract, 10.0 g/L; KH2PO4, 2.0 g/L, pH5.0) at 30°C.

*S. cerevisiae *wild-type, deletion mutant and GST-fusion ORF overexpression strains were obtained through Open Biosystems (Huntsville, AL). *S. cerevisiae *strains were cultured in CM medium with 2% glucose for wild-type and *S. cerevisiae *deletion mutants. CM medium with 2% glucose minus uracil was used for *S. cerevisiae *GST-over expressing strains, and 2% galactose was used to induce the GST-fusion strains. CM broth with glucose and CM broth with glucose minus uracil were purchased from Teknova Inc. (Hollister, CA) (C8000 and C8140 respectively).

Plasmid-containing strains were routinely grown with antibiotics at the following concentrations (μg/mL): kanamycin, 50 for *E. coli *and 200 for ZM4; tetracycline, 10 for *E. coli *and 20 for ZM4; gentamicin, 10 for *E. coli; *and G418, 200 for *S. cerevisiae *YKO deletion mutants. Bacterial growth was monitored using the Bioscreen C automated microbiology growth curve analysis system using 600_nm _filter (Growth Curves USA, Piscataway, NJ).

### PCR and DNA manipulations

Genomic DNA from *Z. mobilis *was isolated using a Wizard Genomic DNA purification kit (Promega, Madison, WI). The QIAprep Spin Miniprep and HiSpeed Plasmid Midi kits (Qiagen, Valencia, CA) were used for plasmid isolation. PCR, restriction enzyme digestion, ligation, cloning, and DNA manipulations were following standard molecular biology approaches as described previously [34] and sequencing was conducted using BigDye Terminator v3.1 cycle sequencing chemistry (Applied Biosystems Inc, Foster City, CA) on a 48-capillary 3730 DNA Analyzer sequencer (Applied Biosystems Inc).

### AcR strain *hfq *insertional mutant construction

An *hfq *insertion mutant was created using the pKnock-Km suicide plasmid system [26,31] in a *Z. mobilis *acetate tolerant strain (AcR) background and the resulting strain designated as AcRIM0347. Briefly, a 262-bp internal DNA fragment of the *Z. mobilis hfq *gene (ZMO0347) was amplified by PCR using primers hfq_MF and hfq_MR (Table 1), and ligated into pKnock-Km using Fast-Link™ DNA Ligation Kit (Epicentre). The plasmid was designated as pKm-0347, which was then electroporated into *E. coli *WM3064. The pKm-0347 plasmid from *E. coli *WM3064 was verified by PCR and Sanger sequencing analysis. AcR and *E. coli *WM3064 (pKm-0347) cells were mixed and plated onto RM agar plates with 100 μg/mL DAP and 50 μg/mL kanamycin for conjugation. The cells were incubated at 30°C overnight and then subcultured on RM agar plates with 50 μg/mL kanamycin in the absence of DAP. Putative conjugants were then screened by PCR using primers hfq_OCF and hfq_OCR (Table 1). Wild-type AcR has a 1,050-bp PCR product and *hfq *mutant candidates had a 2.9-kb PCR product. Presumptive positive PCR products from mutant clones were confirmed by Sanger sequencing analysis.

### Construction of a Gateway vector for ZMO0347 overexpression and mutant complementation

Construction of *hfq *Gateway^® ^entry vector and new destination vector termed pBBR3DEST42 was carried out as previously described [35], except that we used pBBRMCS-3 containing the tetracycline resistance cassette in this study. pBBR3DEST42 was used for *hfq *expression and the resulting vector designated p42-0347. Briefly, DNA for the target gene was amplified via PCR using AcR genomic template DNA and the hfq_CF and hfq_CR primers (Table 1). PCR products were then cloned into Gateway^® ^entry clone pDONR221 using BP Clonase II enzyme mix following the manufacturer's protocol (Invitrogen), transformed into chemically competent DH5α cells (Invitrogen), and plated onto LB with appropriate antibiotic selection. The identity of insert DNA was confirmed by DNA sequence analysis using the M13 forward and reverse primers (Integrated DNA Technologies, Inc., Coralville, IA). The confirmed entry clone vector was then recombined with destination vector pBBR3DEST42 using LR Clonase II enzyme mix (Invitrogen) to create the expression vector, essentially as described previously [35]. The resulting expression plasmid was designed as p42-0347. The plasmid construct p42-0347 was confirmed by DNA sequence analysis.

ZMO0347 overexpression strain ZM4 (p42-0347) and *hfq *complemented mutant strain AcRIM0347 (p42-02347) were generated by conjugation. Briefly, *Z. mobilis *(ZM4 or AcRIM0347) cells were mixed with *E. coli *WM3064 (p42-0347) cells, plated onto RM agar plates with 100 μg/mL DAP and 10 μg/mL tetracycline for conjugation. The cells were incubated at 30°C overnight and then subcultured on RM agar plates with 10 μg/mL tetracycline in the absence of DAP. The tetracycline resistant conjugants were confirmed to be correct using PCR and Sanger sequence analysis.

## Authors' contributions

SY and SDB designed the experiment, analyzed the data and wrote the manuscript. SY constructed the plasmid pBBR3DEST42 and mutant strains and performed the Bioscreen assays. DAP and TSL constructed the expression vector p42-0347 and carried out the Western-blot. All authors read and approved the final manuscript.

## Supplementary Material

Additional file 1 **PPT The comparison of *Z. mobilis *Hfq protein with homologues from other species**. Domain and motif sites of *Z. mobilis *Hfq (A), *E. coli *Hfq (B), *S. cerevisiae *Sm B (D), and *S. cerevisiae *Lsm1 (E) proteins based on NCBI BlastP result as well as the alignment for some bacterial *hfq *homologues (C) using ClustalW 2 http://www.ebi.ac.uk/Tools/clustalw2/index.html. Residues that are identical across the species are indicated by "*", and residues that are not identical but conserved in function across the species are indicated by ":". Click here for file

Additional file 2 **PPT Map of plasmid vector pBBR3DEST42. The vector map of pBBR3DEST42 plasmid constructed to analyze gene over-expressing and complementation**. Tc(R): Tetracycline resistance gene *tet*; Cm: chloramphenicol resistance gene *cat*. *att*R1 and *att*R2 are recombination sites allowing recombinational cloning of the gene of interest from an entry clone; ccdB is *ccdB *gene allowing negative selection of expression clones. Click here for file

Additional file 3 **PPT Lsm proteins in *S. cerevisiae *are involved in multiple inhibitor tolerance**. *S. cerevisiae *strains were grown in CM with 2% glucose (CM + glucose) for wild-type BY4741 and the deletion mutants, CM with 2% glucose and 2% galactose minus uracil (CM + glucose + 2% galactose) for GST overexpression strains. Five-μL culture was then transferred into 250-μL CM broth in the Bioscreen plate. The growth differences of different deletion mutant strains were monitored by Bioscreen (Growth Curves USA, NJ) in CM + glucose at pH 5.5 (A), CM + glucose with 305 mM NaCl, pH 5.5 (B), 305 mM NaAc, pH 5.5 (C), 305 mM NH_4_OAc, pH 5.5 (D), and 305 mM KAc, pH 5.5 (E), 0.75 g/L vanillin, pH 5.5 (F), 1.5 g/L furfural, pH 5.5 (G), and 1.5 g/L HMF, pH 5.5 (H). The growth differences of different GST-over-expressing strains were monitored by Bioscreen (Growth Curves USA, NJ) in CM + glucose + 2% galactose at pH 5.5 (I), CM + glucose + 2% galactose with 305 mM NaCl, pH 5.5 (J), 305 mM NaAc, pH 5.5 (K), 305 mM NH_4_OAc, pH 5.5 (L), 305 mM KAc, pH 5.5 (M), 0.75 g/L vanillin, pH 5.5 (N), 1.5 g/L furfural, pH 5.5 (O), and 1.5 g/L HMF, pH 5.5 (P). Strains included in this study are listed in table 1. This experiment has been repeated at least three times with similar result. Click here for file
