# Supplementary material for: The Zymomonas mobilis regulator hfq contributes to tolerance against multiple lignocellulosic pretreatment inhibitors
Source: BMC Microbiol. 2010 May 7;10:135. doi: 10.1186/1471-2180-10-135 (PMC2877685; doi:10.1186/1471-2180-10-135)
Supplement: Additional file 1 — PPT The comparison of Z. mobilis Hfq protein with homologues from other species. Domain and motif sites of Z. mobilis Hfq (A), E. coli Hfq (B), S. cerevisiae Sm B (D), and S. cerevisiae Lsm1 (E) proteins based on NCBI BlastP result as well as the alignment for some bacterial hfq homologues (C) using ClustalW 2 http://www.ebi.ac.uk/Tools/clustalw2/index.html. Residues that are identical across the species are indicated by "*", and residues that are not identical but conserved in function across the species are indicated by ":". [file 1471-2180-10-135-S1.PPT]

## Slide 1
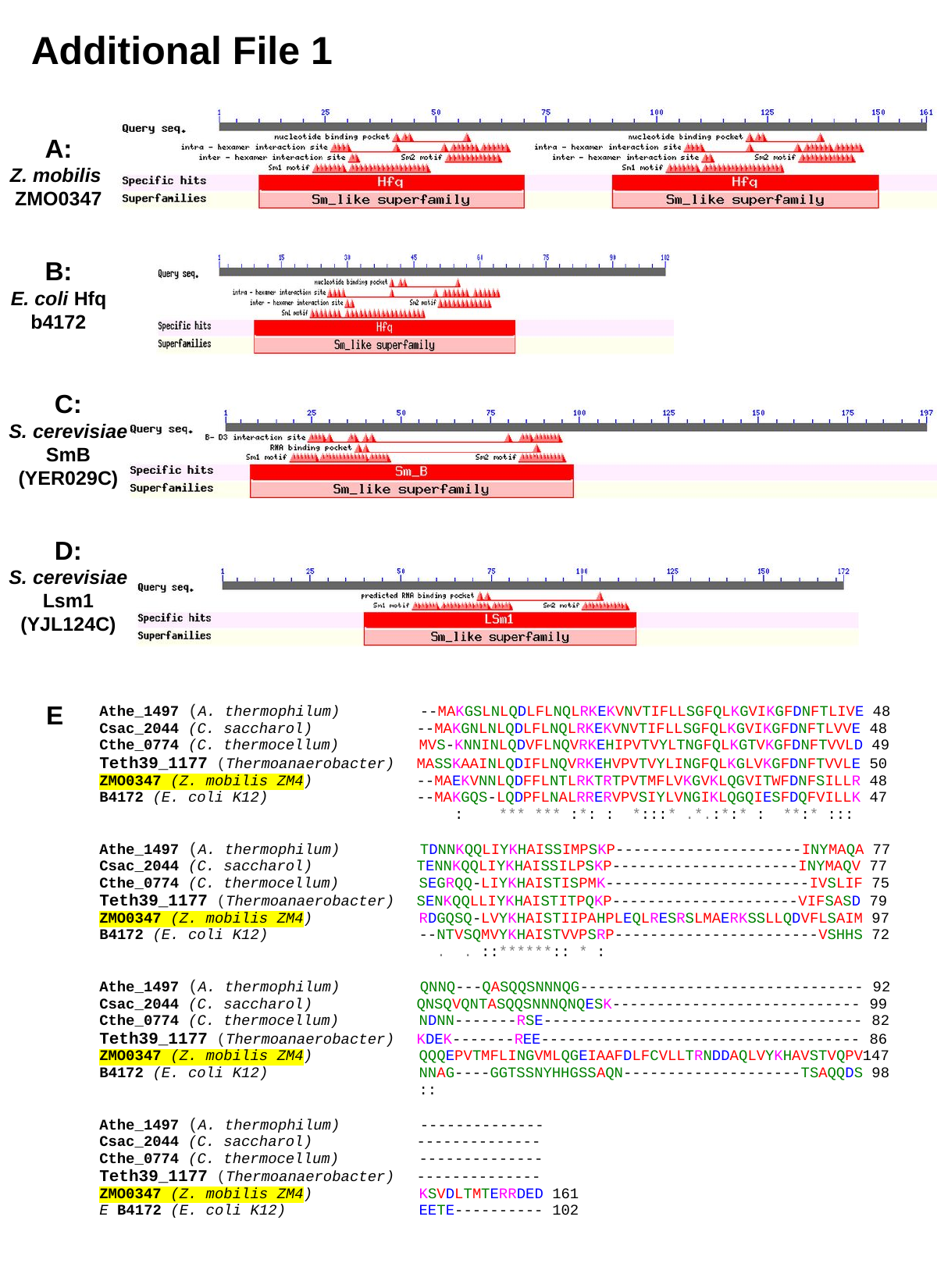

Additional File 1
A:
Z. mobilis
ZMO0347
B:
E. coli Hfq
b4172
C:
S. cerevisiae SmB
(YER029C)
D:
S. cerevisiae Lsm1
(YJL124C)
E
