# Supplementary figures and images for: The Zymomonas mobilis regulator hfq contributes to tolerance against multiple lignocellulosic pretreatment inhibitors
Source: BMC Microbiol. 2010 May 7;10:135. doi: 10.1186/1471-2180-10-135 (PMC2877685; doi:10.1186/1471-2180-10-135)

## Slide 1
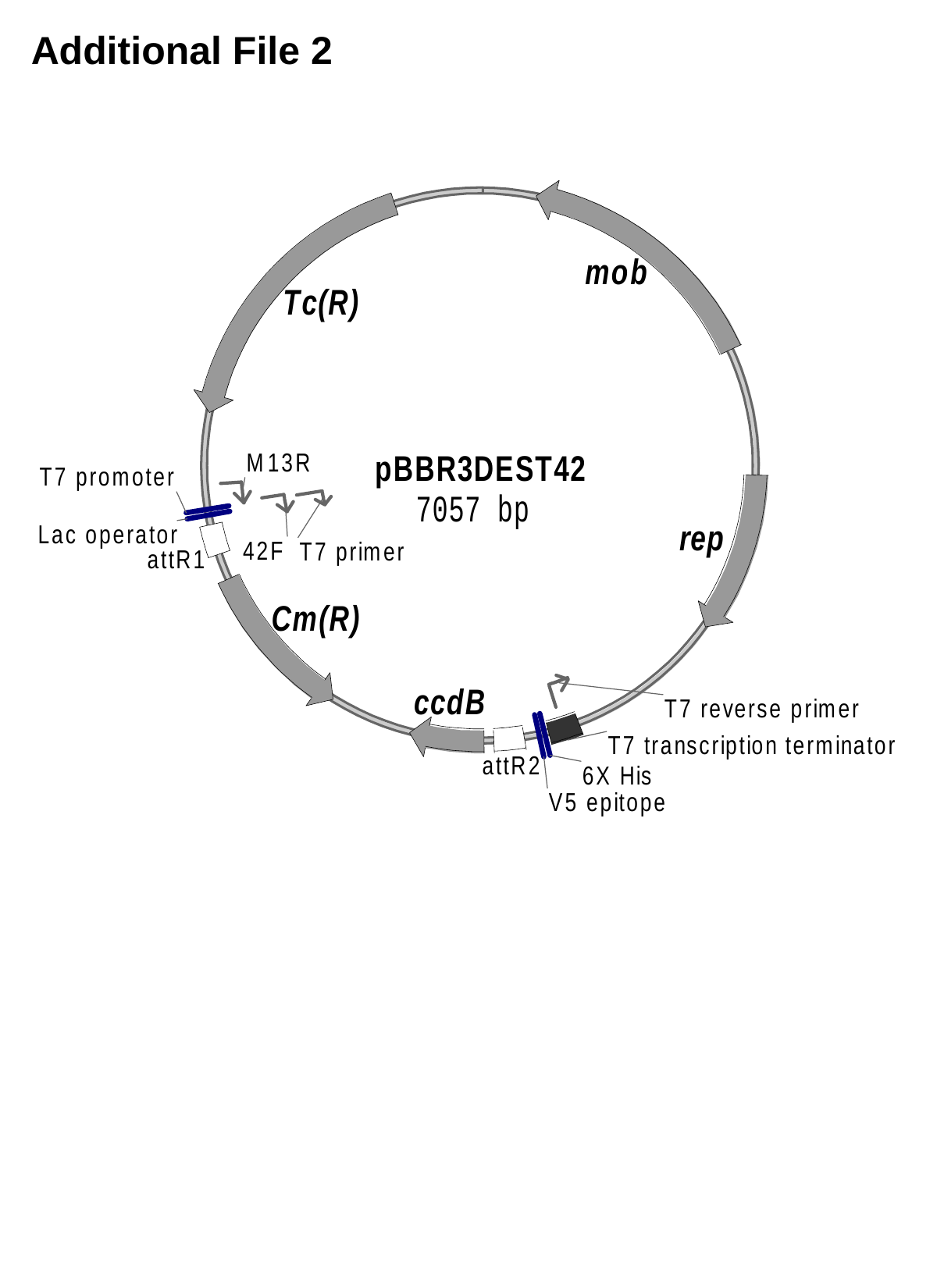

Additional File 2

Supplement: Additional file 2 — PPT Map of plasmid vector pBBR3DEST42. The vector map of pBBR3DEST42 plasmid constructed to analyze gene over-expressing and complementation. Tc(R): Tetracycline resistance gene tet; Cm: chloramphenicol resistance gene cat. attR1 and attR2 are recombination sites allowing recombinational cloning of the gene of interest from an entry clone; ccdB is ccdB gene allowing negative selection of expression clones. [file 1471-2180-10-135-S2.PPT]

## Slide 1
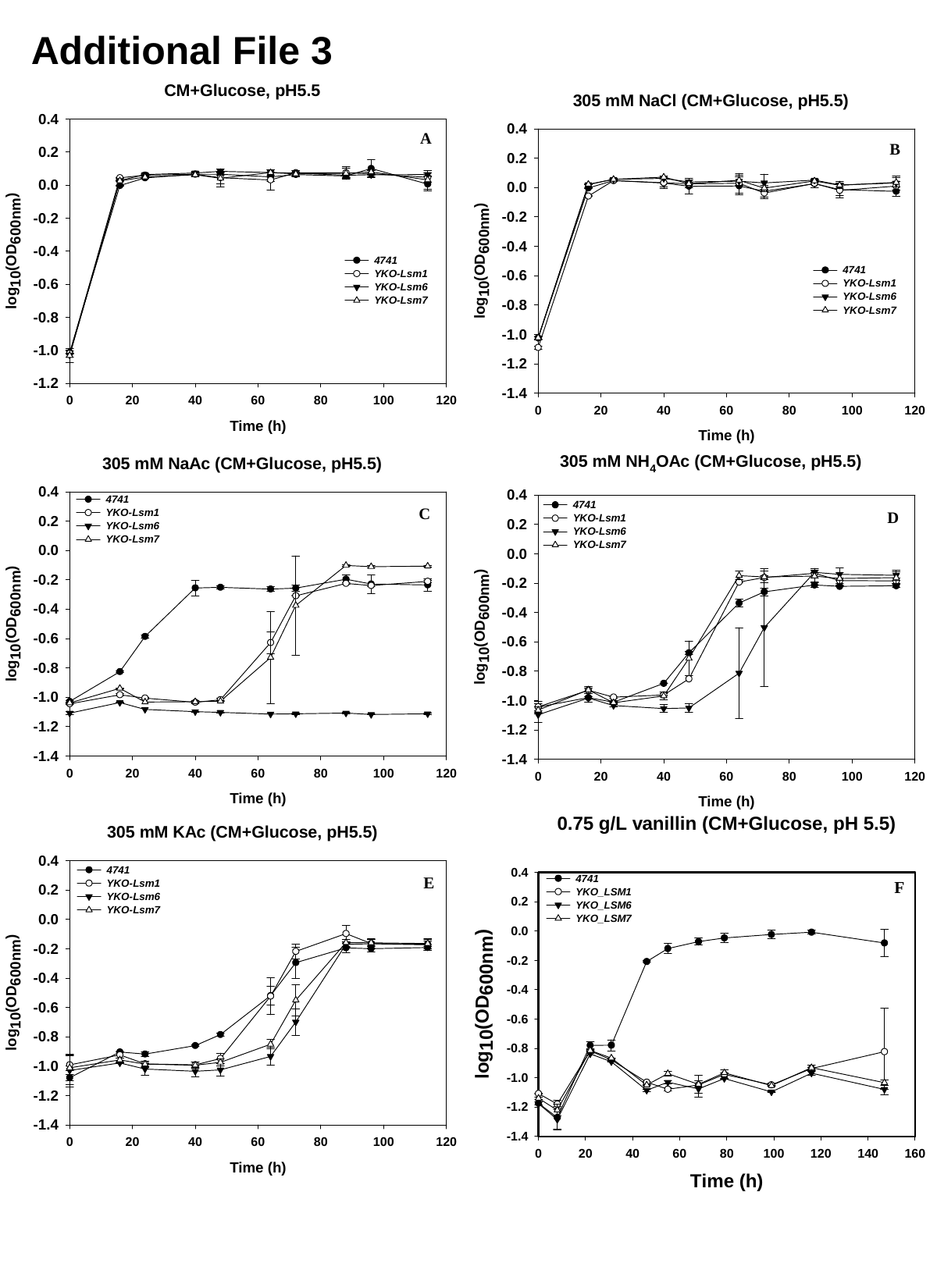

Additional File 3

## Slide 2
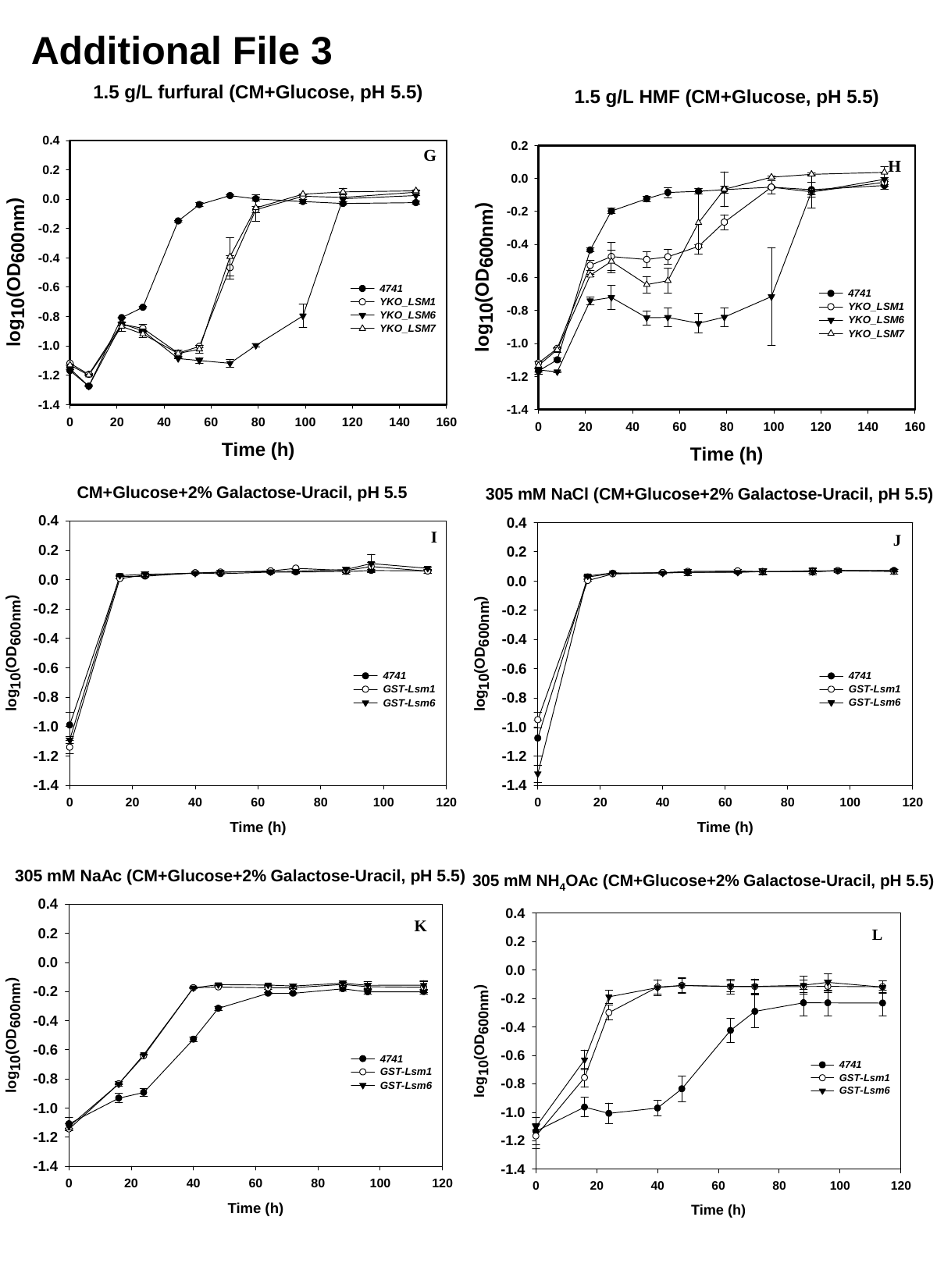

Additional File 3

## Slide 3
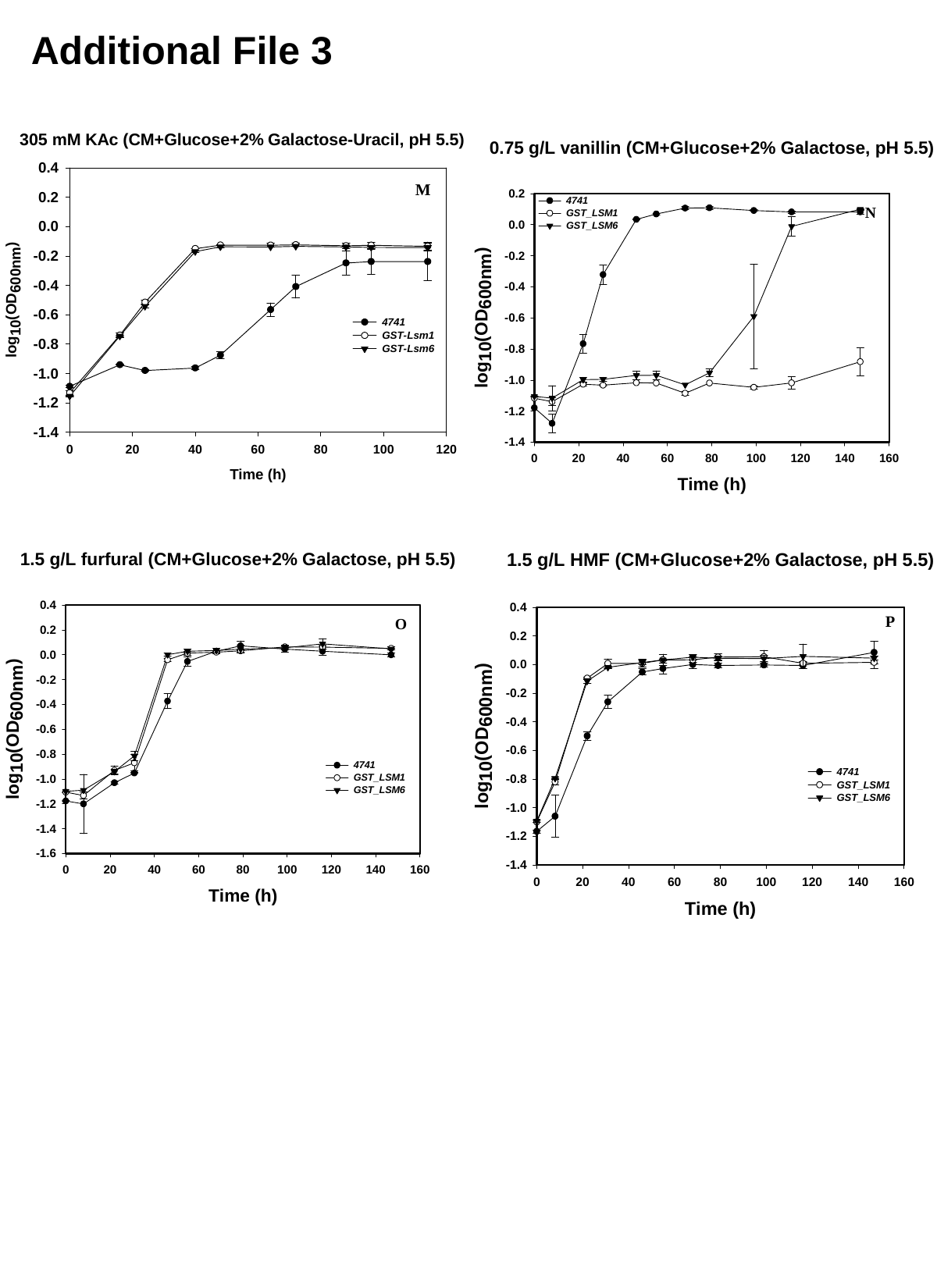

Additional File 3

Supplement: Additional file 3 — PPT Lsm proteins in S. cerevisiae are involved in multiple inhibitor tolerance. S. cerevisiae strains were grown in CM with 2% glucose (CM + glucose) for wild-type BY4741 and the deletion mutants, CM with 2% glucose and 2% galactose minus uracil (CM + glucose + 2% galactose) for GST overexpression strains. Five-μL culture was then transferred into 250-μL CM broth in the Bioscreen plate. The growth differences of different deletion mutant strains were monitored by Bioscreen (Growth Curves USA, NJ) in CM + glucose at pH 5.5 (A), CM + glucose with 305 mM NaCl, pH 5.5 (B), 305 mM NaAc, pH 5.5 (C), 305 mM NH4OAc, pH 5.5 (D), and 305 mM KAc, pH 5.5 (E), 0.75 g/L vanillin, pH 5.5 (F), 1.5 g/L furfural, pH 5.5 (G), and 1.5 g/L HMF, pH 5.5 (H). The growth differences of different GST-over-expressing strains were monitored by Bioscreen (Growth Curves USA, NJ) in CM + glucose + 2% galactose at pH 5.5 (I), CM + glucose + 2% galactose with 305 mM NaCl, pH 5.5 (J), 305 mM NaAc, pH 5.5 (K), 305 mM NH4OAc, pH 5.5 (L), 305 mM KAc, pH 5.5 (M), 0.75 g/L vanillin, pH 5.5 (N), 1.5 g/L furfural, pH 5.5 (O), and 1.5 g/L HMF, pH 5.5 (P). Strains included in this study are listed in table 1. This experiment has been repeated at least three times with similar result. [file 1471-2180-10-135-S3.PPT]
